# Supplementary material for: Ultrafast in-gel detection by fluorescent super-chelator probes with HisQuick-PAGE
Source: Commun Biol. 2020 Mar 20;3:138. doi: 10.1038/s42003-020-0852-1 (PMC7083852; doi:10.1038/s42003-020-0852-1)
Supplement: Supplementary file 1 — Reporting Summary [file 42003_2020_852_MOESM1_ESM.pdf]

nature research

Corresponding author(s): Prof. Dr. Robert Tampé
Goethe University Frankfurt
Institute of Biochemistry, Biocenter
Max-von-Laue-Str. 9
60438 Frankfurt/M.

Last updated by author(s): Nov 22, 2019

Reporting Summary

Nature Research wishes to improve the reproducibility of the work that we publish. This form provides structure for consistency and transparency in reporting. For further information on Nature Research policies, see Authors & Referees and the Editorial Policy Checklist.

Statistics

For all statistical analyses, confirm that the following items are present in the figure legend, table legend, main text, or Methods section.

n/a	Confirmed	
☒	☐	The exact sample size (*n*) for each experimental group/condition, given as a discrete number and unit of measurement
☒	☐	A statement on whether measurements were taken from distinct samples or whether the same sample was measured repeatedly
☒	☐	The statistical test(s) used AND whether they are one- or two-sided<br>*Only common tests should be described solely by name; describe more complex techniques in the Methods section.*
☒	☐	A description of all covariates tested
☒	☐	A description of any assumptions or corrections, such as tests of normality and adjustment for multiple comparisons
☒	☐	A full description of the statistical parameters including central tendency (e.g. means) or other basic estimates (e.g. regression coefficient) AND variation (e.g. standard deviation) or associated estimates of uncertainty (e.g. confidence intervals)
☒	☐	For null hypothesis testing, the test statistic (e.g. *F*, *t*, *r*) with confidence intervals, effect sizes, degrees of freedom and *P* value noted<br>*Give P values as exact values whenever suitable.*
☒	☐	For Bayesian analysis, information on the choice of priors and Markov chain Monte Carlo settings
☒	☐	For hierarchical and complex designs, identification of the appropriate level for tests and full reporting of outcomes
☒	☐	Estimates of effect sizes (e.g. Cohen's *d*, Pearson's *r*), indicating how they were calculated

Our web collection on statistics for biologists contains articles on many of the points above.

Software and code

Policy information about availability of computer code

Data collection	N/A
Data analysis	N/A

For manuscripts utilizing custom algorithms or software that are central to the research but not yet described in published literature, software must be made available to editors/reviewers. We strongly encourage code deposition in a community repository (e.g. GitHub). See the Nature Research guidelines for submitting code & software for further information.

Data

Policy information about availability of data

All manuscripts must include a data availability statement. This statement should provide the following information, where applicable:

- Accession codes, unique identifiers, or web links for publicly available datasets
- A list of figures that have associated raw data
- A description of any restrictions on data availability

The authors declare that all data supporting the findings of this study are available from the corresponding author upon request.

Field-specific reporting

Please select the one below that is the best fit for your research. If you are not sure, read the appropriate sections before making your selection.

☒ Life sciences ☐ Behavioural & social sciences ☐ Ecological, evolutionary & environmental sciences

For a reference copy of the document with all sections, see nature.com/documents/nr-reporting-summary-flat.pdf

Life sciences study design

All studies must disclose on these points even when the disclosure is negative.

Sample size	n/a
Data exclusions	No data in the present study was excluded.
Replication	Experiments shown in the present study were performed at least three times and a representative example was chosen for publication.
Randomization	Randomization or statistics were not relevant to the present study. The findings about the shown method require neither statistical evaluations nor experimental randomization.
Blinding	Blinding was not relevant to the present format, because the data could not be biased by an open study.

Reporting for specific materials, systems and methods

We require information from authors about some types of materials, experimental systems and methods used in many studies. Here, indicate whether each material, system or method listed is relevant to your study. If you are not sure if a list item applies to your research, read the appropriate section before selecting a response.

Materials & experimental systems

n/a	Involved in the study	
☐	☒	Antibodies
☐	☒	Eukaryotic cell lines
☒	☐	Palaeontology
☒	☐	Animals and other organisms
☒	☐	Human research participants
☒	☐	Clinical data

Methods

n/a	Involved in the study	
☒	☐	ChIP-seq
☒	☐	Flow cytometry
☒	☐	MRI-based neuroimaging

Antibodies

Antibodies used	abcam: Anti-6X His tag® antibody (HRP), polyclonal, rabbit, ab1187 (LOT: GR300288-7#)<br>Sigma Aldrich: Monoclonal Anti-polyHistidine antibody produced in mouse, H1029 (LOT: 097M4894V)<br>Merck: His•Tag® Monoclonal Antibody, mouse, 70796 (LOT: 3127944)<br>Cell Signaling Technology: DYKDDDDK Tag (D6W5B) Rabbit mAb #14793 (LOT: 4)<br>Sigma Aldrich: Goat Anti-Rabbit IgG Antibody, (H+L) HRP conjugate # AP307P (LOT: 3136001)<br>Sigma Aldrich: Anti-Mouse IgG (Fc specific)–Peroxidase antibody produced in goat A2554 (LOT: 128M4771V)
Validation	Corresponding comprehensive validation information, further publications and protocols about the present antibodies used in this study are available at the manufactures homepage. Furthermore, the present data validates the functionality.

Eukaryotic cell lines

Policy information about cell lines

Cell line source(s)	E. coli : One Shot BL21 (DE3), ThermoFisher Scientific, Invitrogen<br>S. cerevisiae: Gift by Dr. Peter Koetter (EUROSCARF)<br>S. frugiperda: TriEx Sf9 Cells, Merck, Novagen<br>H. sapiens: HeLa Kyoto, DSMZ, Leibniz-Institut
Authentication	Authentication procedures are stated by the suppliers.
Mycoplasma contamination	Mycoplasma contamination tests were carried out regularly, following established guidelines. Uphoff, C. C. & Drexler, H. G. Detection of Mycoplasma Contamination in Cell Cultures. Curr. Protoc. Mol. Biol. 106, 28.4:28.4.1–28.4.14 (2014)

Commonly misidentified lines<br>(See ICLAC register)	n/a
