# Supplementary material for: Ultrafast in-gel detection by fluorescent super-chelator probes with HisQuick-PAGE
Source: Commun Biol. 2020 Mar 20;3:138. doi: 10.1038/s42003-020-0852-1 (PMC7083852; doi:10.1038/s42003-020-0852-1)
Supplement: Supplementary file 2 — Supplementary Information [file 42003_2020_852_MOESM2_ESM.pdf]

## Supplemental Information

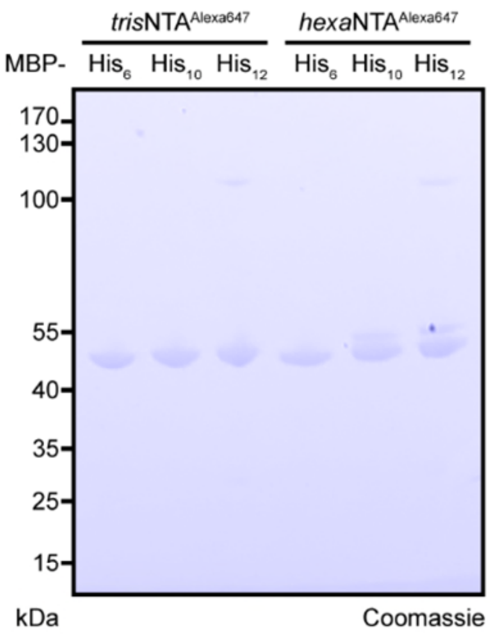


**Supplementary Figure 1 | Characterization of fluorescent chelator probes for SDS-PAGE protein detection.** Coomassie staining of non-reducing SDS-PAGE analysis of various sized histidine tags ($His_n$) on 2 µg MBP (45 pmol) incubated with 450 nM (7 pmol) of *tris*NTA$^{Alexa647}$ or *hexa*NTA$^{Alexa647}$. A slight upshift of the reference proteins MBP-$His_{10,12}$ is visible and confirming the labeling by *hexa*NTA$^{Alexa647}$ (Fig. 3a).

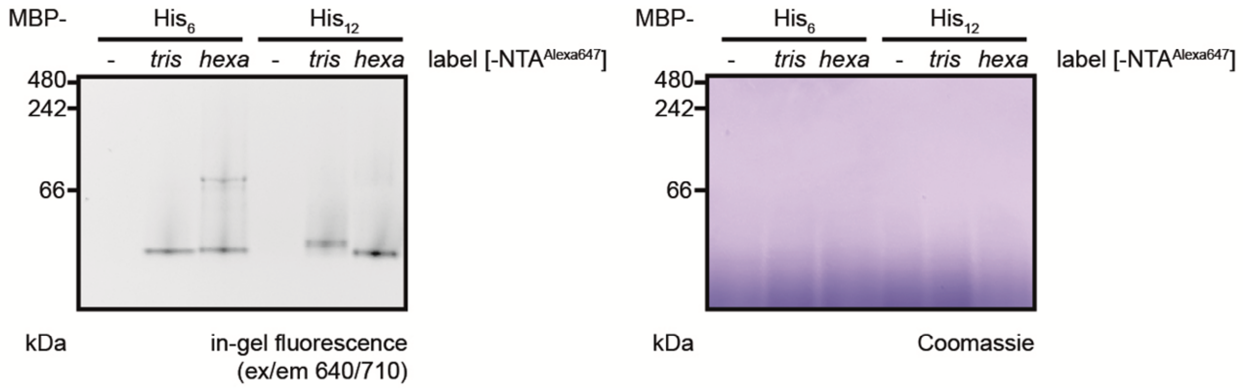


**Supplementary Figure 2 | Characterization of fluorescent chelator probes for blue native PAGE protein detection.** In-gel fluorescence and Coomassie staining of $His_6$- and $His_{12}$-tagged MBP (2 µg, 45 pmol) incubated with 450 nM (7 pmol) of *tris*$NTA^{Alexa647}$ or *hexa*$NTA^{Alexa647}$.
